# Newly emerging metronidazole-resistant *Clostridioides difficile* PCR ribotype 955 identified in Poland, 2021 to 2023 but not in Czechia, 2012 to 2023 and Slovakia, 2015 to 2023

**DOI:** 10.2807/1560-7917.ES.2025.30.21.2400675

**Published:** 2025-05-29

**Authors:** Jaroslava Zikova, Klaudia Szarek, Monika Kabała, Dorota Wultańska, Natalia Frankowska, Adam Iwanicki, Krzysztof Hinc, Anna Mucha, Jolanta Komarnicka, Anna Jagielska, Ed Kuijper, Marcela Krutova, Hanna Pituch

**Affiliations:** 1 Department of Medical Microbiology, Charles University Second Faculty of Medicine and Motol University Hospital, Prague, Czechia; 2 European Society for Clinical Microbiology and Infectious Diseases (ESGMID) study group for *Clostridioides difficile* (ESGCD), Basel, Switzerland; 3 Department of Medical Microbiology, Faculty of Medical Sciences in Katowice, Medical University of Silesia, Katowice, Poland; 4 Department of Medical Microbiology, Medical University of Warsaw, Warsaw, Poland; 5 Division of Molecular Bacteriology, Medical University of Gdańsk, Gdańsk, Poland; 6 Intercollegiate Faculty of Biotechnology, University of Gdańsk, Gdańsk, Poland; 7 Department of Pulmonology with Oncology and Chemotherapy, Center of Pulmonology and Thoracic Surgery in Bystra, Poland; 8 Laboratory of Clinical Microbiology, University Centre for Laboratory Diagnostics, Medical University of Gdańsk Clinical Centre, Gdańsk, Poland; 9 Department of Social Medicine and Public Health, Medical Faculty, Medical University of Warsaw, Warsaw, Poland; 10 National Expertise Centre for *Clostridioides difficile* infections, Leiden University Center for Infectious Diseases, Leiden and Centre for Infectious Disease Control (CIb), National Institute for Public Health and the Environment (RIVM), Bilthoven, Netherlands

**Keywords:** Surveillance, Tyr130Ser, Leu155Ile, heme-dependent, fluoroquinolones, *erm*B, *nim*B, *aac*(6')-*aph*(2”)

## Abstract

**Background:**

On 29 January 2024, the European Centre for Disease Prevention and Control distributed an alert about a metronidazole-resistant *Clostridioides difficile* outbreak of PCR ribotype (RT) 955 in England.

**Aim:**

We aimed to investigate the presence of RT955 in Czech, Slovak and Polish *C. difficile* isolates and evaluate different culture media for detecting its metronidazole resistance.

**Methods:**

Isolates with binary toxin genes identified as ‘unknown’ by the WEBRIBO PCR ribotyping database up to 2023 were re-analysed after adding the RT955 profile to the database. The RT955 isolates were characterised by whole genome sequencing and tested for susceptibility to 15 antimicrobials.

**Results:**

We did not find RT955 in Czech (n = 6,661, 2012–2023) and Slovak (n = 776, 2015–2023) isolates, but identified 13 RT955 cases (n = 303, 2021–2023) in three hospitals in Poland. By whole genome multilocus sequence typing, 10 isolates clustered into one clonal complex including a sequence of United Kingdom strain ERR12670107, and shared similar antimicrobial resistance genes/mutations. All 13 isolates were resistant to ciprofloxacin/moxifloxacin, erythromycin/clindamycin and ceftazidime. All isolates had a mutation in the *nim*B gene promoter and in NimB (Tyr130Ser and Leu155Ile). The metronidazole resistance was detected in all isolates using brain-heart-infusion agar supplemented with haemin and Chocolate agar. Results were discrepant with the European Committee on Antimicrobial Susceptibility Testing-recommended Fastidious anaerobe agar and Brucella blood agar.

**Conclusion:**

The identification of clonally related haem-dependent metronidazole-resistant *C. difficile* RT955 in multiple hospitals indicates a need for prospective surveillance to estimate its prevalence in Europe.

**Key public health message**

**What did you want to address in this study and why?**

*Clostridioides difficile* is the main cause of hospital-acquired diarrhoea. In December 2023, a new so-called hypervirulent *C. difficile* (type 955) emerged in the United Kingdom (UK). It caused increased mortality of 22.9% and was resistant to metronidazole, one of the drugs used to treat *C. difficile* infection. Since similar strains have rapidly spread to other countries, we searched for type 955 in Czech, Slovak and Polish collections of *C. difficile* isolates.

**What have we learnt from this study?**

We found *C. difficile* type 955 in three hospitals in Poland. The Polish strains were closely related to the British strain, but it is not clear whether the origin of type 955 was in Poland or the UK. Using culture media with a specific composition, we identified all strains as resistant to metronidazole.

**What are the implications of your findings for public health?**

We recommend performing national studies to estimate the occurrence of *C. difficile* type 955 in healthcare facilities, especially when metronidazole is still used for *C. difficile* infection treatment.

## Background

*Clostridioides* *difficile* is the leading cause of healthcare-associated diarrhoea, *C. difficile* infection (CDI). The epidemiology of CDI has changed dramatically after the emergence and global spread of hypervirulent PCR ribotype (RT) 027 [1]. The use of molecular typing techniques, such as PCR ribotyping and whole genome sequencing in CDI surveillance enhanced the identification of potential outbreaks and improved the monitoring of the spread of epidemic ribotypes [2]. Although PCR ribotyping is the most frequently applied typing method for *C. difficile* [2] and is also recommended by the European Centre for Disease Prevention and Control (ECDC), only a few laboratories have a complete and regularly updated database to recognise all PCR ribotypes.

Since 2003, surveillance studies have revealed variants of the ‘hypervirulent’ *C. difficile* RT027 in southern and eastern Europe. Ribotype 176 has caused outbreaks in Croatia, Czechia, Poland and Slovakia [3-6] and RT181 in Romania and Greece [7,8]. Similar to *C. difficile* RT027, these new ribotypes belonged to sequence type 1 and clade 2, carried genes for binary toxin, and had an 18 bp deletion in the *tcdC* gene at position 330–347 and a single nucleotide deletion at position 117 [8,9].

On 29 January 2024, the ECDC reported through EpiPulse, an online portal for European public health authorities and partner organisations, a notification of a *C. difficile* outbreak of the newly found RT955 in England. The United Kingdom (UK) Health Security Agency (UKHSA) deposited the UK RT955 strain in the National Centre for Bioinformatic Information (NCBI) Sequence Read Archive (SRA), accession number ERR12670107, to provide other laboratories with the opportunity to recognise this strain. In addition, the PCR ribotype profile was made accessible to other laboratories in the WEBRIBO database. Further details on the outbreak were presented in April 2024 at an international conference in Barcelona, Spain [10,11]. In summary, between September 2021 and December 2023, 50 CDI cases (48 unique CDI patients with two recurrences) were identified in the UK [10,11]. Four regions in England were affected by sporadic cases and another region reported two large hospital clusters. Eleven of the 48 patients died in the 30 days following the detection of the infection. The *C. difficile* RT955 isolates belonging to clade 2 carried binary toxin genes were clonal by multiple-locus variable-number tandem-repeat analysis (MLVA) (n = 25) and were closely related by whole genome sequencing (WGS) (n = 48). Antibiotic resistance was reported to moxifloxacin, rifampicin, clindamycin, imipenem and, most importantly, to metronidazole [10,11], the antimicrobial drug still used for the treatment of CDI in clinical practice.

In response to the EpiPulse notification, we searched for this ribotype in the Czech, Slovak and Polish collections of *C. difficile* isolates. We further evaluated different culture media to detect the metronidazole resistance that was reported in UK RT955 isolates.

## Methods

### *Clostridioides difficile* isolates

The *C. difficile* collections used for the RT955 survey included Czech *C. difficile* isolates (n = 6,661) collected between 2012 and 2023 and Slovak isolates (n = 776) collected between 2015 and 2023 during several time-limited surveillance studies. Further, we included Polish isolates (n = 303) from a study entitled “The role of prophages in the virulence of clinical strains of human pathogen *Clostridioides difficile *in vitro and in vivo (acronym PROPHDIFF)” that were collected between 2021 and 2023 in seven hospitals. All *C. difficile* isolates (n > 7,700) were characterised and are stored at Motol University Hospital in Prague, Czechia. The number of isolates and hospitals for each year is given in the Supplementary material.

**Figure 1:**
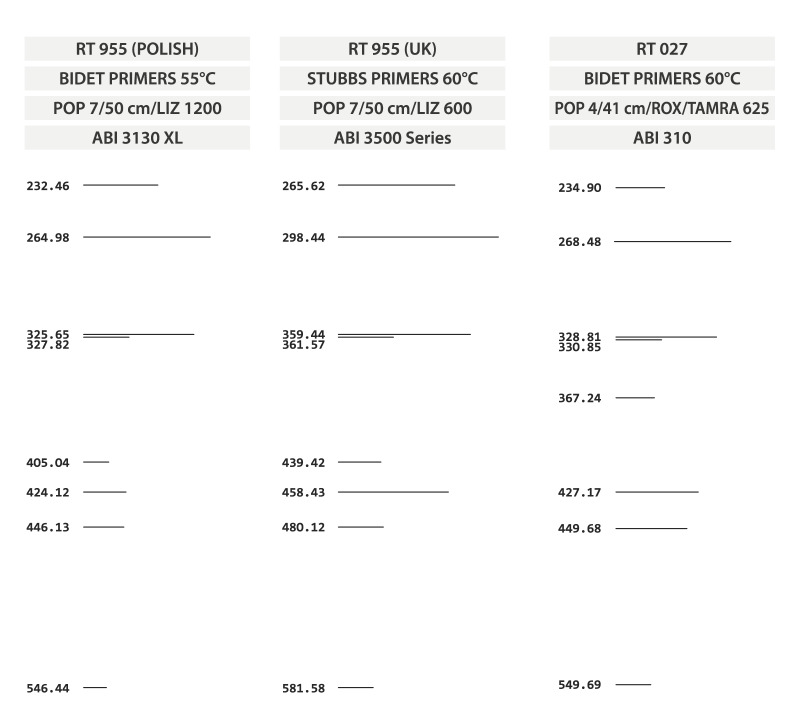
Capillary gel-based electrophoresis PCR ribotyping profiles of *Clostridioides difficile* RT955 and RT027

All *C. difficile* isolates are characterised by multiplex PCR for detection of genes for toxins (*tcd*A-toxin A, *tcd*B-toxin B, *ctd*A and *cdt*B-binary toxin) [12] and by high-resolution capillary gel-based electrophoresis (CE) PCR ribotyping using Bidet et al. and/or Stubbs et al. primers [13-15] that amplify the intergenic spacer region between 16S and 23S rRNA genes. The PCR ribotype was determined using the WEBRIBO database (https://webribo.ages.at). Because the profile of RT955 was added to the WEBRIBO database in 2024 and the RT955 had a binary toxin gene [10,11], we re-analysed the ribotyping profiles of isolates positive for binary toxin genes for which the ribotype had by 2023 not been determined. The CE-PCR ribotyping profiles of RT955 and RT027, including the different fragment sizes as determined by different primer pairs designed by Bidet et al. and Stubs et al. [14,15], are shown in Figure 1.

Profiles as obtained from the WEBRIBO database. Primers by Bidet et al. and Stubbs et al. were used for RT955, primers by Bidet et al. were used for RT027 [14,15].

### Antimicrobial susceptibility testing

Thirteen identified RT955 isolates were tested for antimicrobial susceptibility to 15 antimicrobials using an E-test on Brucella blood agar (cat no. 411968, bioMérieux, France) except for fidaxomicin. Susceptibility to fidaxomicin was tested by agar dilution method using Wilkins–Chalgren agar (cat no. CM0619, Oxoid, UK). Plates were evaluated after 48 h in an anaerobic atmosphere. In addition, we tested susceptibility to metronidazole using E-test on four different batches of Fastidious anaerobe agar with 5% defibrinated horse blood (cat no. PB0225A, Oxoid, UK), as recommended by the European Committee on Antimicrobial Susceptibility Testing (EUCAST) [16], on Chocolate agar with Vitox containing 10 g/L of haemoglobin (cat no. PO5090A, Oxoid, UK) as a commercially available ready-to-use haemin enriched media, and on brain-heart-infusion (BHI) agar (cat no. CM1136, Oxoid, UK) either with or without 5, 10 and 20 mg/L of bovine and porcine haemin (Sigma Aldrich, catalogue no. H9039 and 51280) [17]. Plates were evaluated after 24 h and 48 h of culture in an anaerobic atmosphere at 36.6 °C (Anaerobic Workstation, Don Whitley Scientific, UK). *Bacteroides fragilis* ATCC 25285 and *C. difficile* RT010 (strain ID: 5863, minimum inhibitory concentration (MIC) to metronidazole 12 mg/L [18], included for metronidazole susceptibility re-testing) were used as controls. The composition of media used for susceptibility testing is available in the Supplement. Multidrug resistance was defined as acquired non-susceptibility to at least one agent in three or more antimicrobial categories [19].

### Whole genome sequencing and bioinformatic analysis

The bacterial DNA was extracted using MasterPure Gram Positive DNA Purification Kit (Biosearch Technologies, UK). The DNA library was prepared using Nextera XT library preparation kit (Illumina, US) and sequenced on NextSeq2000 (Illumina, United States (US), all isolates). In addition, one isolate was sequenced on GridION (Oxford Nanopore Technologies, UK) after library preparation using Rapid barcoding Kit v14 on the flow cell FLO-MIN114 v10.4.1 (both Oxford Nanopore Technologies, UK) to provide a reference genome. Short reads were assembled using SPAdes v 3.15.5. Hybrid assembly from short and long reads was performed using Flye v2.9.1 (long reads assembly), Medaka v1.7.2 (polishing by long reads), and Polypolish v0.5.0 (polishing by short reads). Fasta files were annotated in RAST (https://rast.nmpdr.org).

We determined a multilocus sequence type (MLST) and a core genome multilocus sequence type (cgMLST) from FASTQ data using MLSTFinder v2.0 and cgMLSTFinder v1.1 [20,21]. We performed whole genome multilocus sequence typing (wgMLST) and constructed a minimum spanning tree (MST) using BioNumerics (v8.1, bioMérieux, France), including the sequence of the UK RT955 strain (NCBI SRA accession number ERR12670107). Acquired antimicrobial resistance genes were detected using ResFinder v4.1 after uploading fastq data, with the default setting, and ‘other species’ selection [22] and then searched in annotated assemblies. The mutations in the *gyr*A gene for fluoroquinolone resistance [23], in the *rpo*B gene for rifampicin resistance [24], in the *pbp*1 and *pbp*3 for resistance to cephalosporines [25], in the *nim*B gene and its promoter [17] and in the *hsm*A gene for metronidazole resistance [26] were searched through alignment to *C. difficile* CD196, GCF_021378415.1 using Geneious software v2021.0.3 (Dotmatics, US. We assessed the presence of pCD-METRO [27] by mapping short reads to metronidazole-resistant *C. difficile* RT020, IB136; GCF_900696735.1. We searched for deletions in the *tcd*C gene by alignment to *C. difficile* CD630, GCF_000009205.2 (Geneious software v2021.0.3, Dotmatics, US). The whole genome comparison was visualised using EasyFig v2.2.5 to identify the genomic context with antimicrobial resistance determinants.

### Statistical analysis

Overall mortality and recurrence rates between Polish and UK patients with RT955 CDI were compared using Fisher’s exact test.

## Results

### *Clostridioides difficile* ribotype 955 infection cases

We did not find RT955 in Czech (n = 6,661) and Slovak (n = 776) *C. difficile* isolates. Among the 303 Polish isolates, we identified a total of 13 isolates belonging to RT955 (Figure 1). These 13 Polish RT955 CDI cases (individual patients) were identified between September 2021 and April 2023 in three different hospitals (cities). The patients’ data are summarised in Table 1. When we compared the demographic and clinical data of the 13 Polish patients from this study with 48 UK CDI cases [10,11], the patients had a similarly wide range of ages, including age categories not traditionally associated with CDI (six patients younger than 45 years in the UK [10,11] and four Polish patients younger than 65 years, one of whom was in their 30s). There were both healthcare- and community-associated cases in both studies [10,11]. The reported overall mortality in RT955 CDIs was higher in Polish RT955 CDI patients (30.8% vs 22.9%) but without statistical significance (p > 0.05). The recurrence rate was higher in Polish patients (38.5% vs 4.0%; p = 0.01). Information on CDI treatment was not available.

**Table 1:** *Clostridioides difficile* ribotype 955 infection, patient characteristics, Poland, 2021–2023 (n = 13)

Patient characteristics	n	%
Median age in years (range)	75 (35–98)	
Patient < 65 years	4	30.8
Male	8	61.5
Female	5	38.5
Hospitalised in previous 3 months	10	76.2
No antibiotics or PPIs before CDI	5	38.5
Healthcare-associated CDI	7	53.9
Community-associated CDI	1	7.7
Recurrent CDI	5	38.5
Severe CDI	6	46.2
Death	4	30.8

CDI: *Clostridioides difficile* infection; PPI: proton pump inhibitors.

### Genetic relatedness of *Clostridioides difficile* ribotype 955 isolates

**Figure 2:**
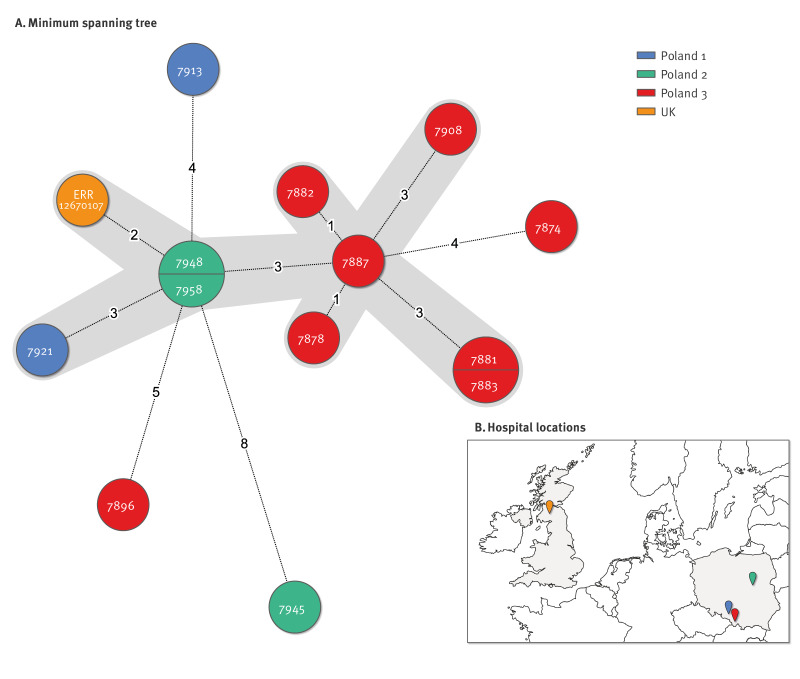
Minimum spanning tree of *Clostridioides difficile* ribotype 955 isolates and localisation of hospitals where the cases were identified, Poland 2021–2023 (n = 13)

All *C. difficile* RT955 isolates belonged to ST1 and clade 2, carried the binary toxin, and had a a 1 bp deletion in the *tcdC* gene at position 117 and an 18 bp deletion in the *tcd*C gene at position 330–347. By cgMLST Finder, all were designated as type 6321. By wgMLST (3,279 loci), we identified one clonal complex (0–3 alleles differences) consisting of nine Polish isolates (Hospital 1: n = 1; Hospital 2: n = 2 and Hospital 3: n = 6) and UK strain ERR12670107 (Figure 2).

The figure includes United Kingdom strain ERR12670107 used for determination of genetic relatedness and search for antimicrobial resistance determinants. The tree was constructed based on whole genome multilocus sequence typing data using Bionumerics v8.1., edited in Affinity Designer v2. The numbers in the circles represent the identification numbers of *C. difficile* PCR ribotype 955 isolates. The numbers on the lines represent the number of allele differences between isolates.

The clonal cluster (0–3 alleles differences) comprised nine Polish isolates derived from three hospitals at a distance of 80 km between Hospitals 1 and 3, around 300 km between Hospitals 2 and 3, and around 400 km between Hospitals 1 and 2. In Hospital 1, only one patient was identified, but this patient has been admitted from another unrelated hospital and had been hospitalised three times in the previous 12 weeks. The two patients in Hospital 2, were hospitalised in two different wards (nephrology and neurosurgery) and developed CDI 8 and 38 days after admission; their hospitalisation overlapped, and they were diagnosed with CDI within 4 days of each other. Both patients had no record of hospitalisation in the previous 12 weeks. The six patients in Hospital 3 were hospitalised in three different wards and diagnosed with CDI in different months. All six patients had been hospitalised elsewhere in the previous 12 months, two patients had recurrent CDI, two patients had been admitted from a long-term care facility and one patient from another hospital. Two of them had diarrhoea and tested positive for CDI on the day of admission.

### Antimicrobial susceptibility testing and resistance determinants

All isolates were phenotypically susceptible to vancomycin, fidaxomicin, amoxicillin/clavulanic acid, tetracycline, ertapenem, imipenem, meropenem and benzylpenicillin.

Phenotypical resistance to ciprofloxacin/moxifloxacin was associated with the presence of *gyr*A_p.Thr82Ile, resistance to rifampicin was associated with *rpo*B_p.Arg505Lys and Val134Ile, resistance to ceftazidime was associated with *pbp*3_p.Val497Leu, and resistance to erythromycin/clindamycin was mediated by the *erm*B gene. Two isolates were resistant to metronidazole on Brucella blood agar, however, all isolates had a T-to-G mutation in the −43 nt position in the promoter region of the *nim*B gene and two substitutions in the *nim*B gene (p.Tyr130Ser, and Leu155Ile). The above mechanisms of resistance were also detected in the UK RT955 genome. Plasmid pCD-METRO and mutations in the haem-responsive *hsm*A gene were absent.

As the detected molecular mechanism of metronidazole resistance is haem-dependent [17], we re-tested metronidazole susceptibility on different culture media containing haemin or haemoglobin (Table 2). The resistance phenotype was observed in all 13 Polish isolates using Chocolate agar and BHI agar supplemented with 5, 10 or 20 mg/L of haemin after 24 h and 48 h. Discrepant phenotypes were observed in two of four batches of Fastidious anaerobe agar when 84.6%, 76.9%, 15.4% and zero isolates were resistant after 24 h and 100%, 100%, 46.2% and 7.7% of isolates were resistant after 48 h, respectively. All tested isolates remained susceptible to metronidazole on BHI agar without haemin after 24 h and 48 h of incubation (Table 2). Supplementary Table S3 provides the MICs in individual isolates. The metronidazole susceptibility MICs did not differ between media with bovine or porcine haemin (data not shown).

**Table 2:** Summary of antimicrobials with detected phenotypic resistance after 48 h of culture and corresponding resistance determinants

Antimicrobials (E-test, agar dilution^a^)	MIC range (mg/L)	Culture media	Breakpoint/ECOFF value (mg/L)	Resistant		Molecular resistance determinant
				n	%	
Vancomycin	0.064–0.5	BBA	> 2^b^	None		None
	0.25–0.5	FAA		None		
Metronidazole	0.25–3	BBA (5 mg/L haemin)	> 2^b^	2	15.4	P*nim*B^G^, *nim*B_p.L155I, Y130S
	3–6	FAA (10 mg/L haemin, batch 1)		13	100	
	1–3	FAA (10 mg/L haemin, batch 2)		6	46.2	
	3–6	FAA (10 mg/L haemin, batch 3)		13	100	
	1–3	FAA (10 mg/L haemin, batch 4)		1	7.7	
	6–8	CA (10 g/L haemoglobin)		13	100	
	4–12	BHI with 5, 10, and 20 mg/L haemin^a^		13	100	
	0.19–0.5	BHI without haemin		None		
Fidaxomicin	0.008–0.0625	WCA	> 0.5^b^	None		None
Erythromycin	> 256	BBA	≥ 8^bc^	13	100	*erm*B
Clindamycin	> 256	BBA	≥ 8^c^	13	100	
Rifampicin	> 32	BBA	NA	13	100	*rpo*B_p.V134I, R505K
Ciprofloxacin	> 32	BBA	≥ 8^c^	13	100	*gyr*A_p.T82I
Moxifloxacin	> 32	BBA	≥ 8^c^	13	100	
Ceftazidime	48– >256	BBA	NA	13	100	*pbp*3_p.V497L
Imipenem	8–12	BBA	≥ 16^c^	None		None
Ertapenem	0.5–2	BBA	≥ 16^c^	None		None
Meropenem	0.38–1.5	BBA	≥ 16^c^	None		None
Amoxicillin/clavulanic acid	0.032–0.38	BBA	≥ 16^c^	None		None
Benzylpenicillin	0.38–1.5	BBA	NA	None		None
Tetracycline	0.016–0.125	BBA	≥ 16^c^	None		None

BBA: Brucella blood agar; BHI: brain-heart-infusion agar; CA: Chocolate agar; ECOFF: epidemiological cut-off; FAA: fastidious anaerobe agar (four different batches); MIC: minimum inhibitory concentration; NA: not available; WCA: Wilkins–Chalgren agar.

^a ^Addition: haemin from bovine, ≥ 90%, Sigma-Aldrich.

^b^ The European Committee on Antimicrobial Susceptibility Testing (EUCAST) ECOFFs [16].

^c^ Clinical and Laboratory Standards Institute (CLSI) breakpoints [38].

When we searched for other acquired resistance genes, we noted the presence of *aac*(6´)-*aph*(2´´) genes. The corresponding phenotypical resistance to amikacin and gentamicin was not tested, since *C. difficile* is inherently resistant to aminoglycosides [28].

The tested antimicrobials and used breakpoints and epidemiological cut-off values (ECOFFs) and a summary of antimicrobial susceptibility testing and detection of resistance mechanisms is shown in Table 2. The individual MICs and results after 24h incubation are summarised in the Supplementary Table S3.

## Discussion

In our retrospective analysis of 7,740 strains collected from three countries in the period from 2012 to 2023, we identified 13 isolates of *C. difficile* RT955 in three hospitals in Poland using an updated database where RT955 was recently included. None of the hospitals encountered an outbreak of CDI due to RT955, but spread of identical strains within and between hospitals occurred.

Using wgMLST, we found a cluster of nine isolates in three different Polish hospitals with an identical resistome, suggesting a clonal spread. However, we did not identify direct transmission between patients on the same ward, thus contaminated hospital environment could play a role in transmission.

Interestingly, the UK strain ERR12670107 clustered with two Polish strains from Hospital 2 (2 alleles difference) and met the proposed adjusted threshold of zero to three allelic differences for outbreak recognition [29]. Whether the origin of RT955 was in Poland or the UK cannot be determined because the first CDI cases of RT955 from both countries were notified in the same month and year (September 2021). A possible explanation for transmission between Poland and the UK could be tourists in need of medical support or the movement of healthcare workers between the two countries.

The genetic relatedness of the UK and Polish strains is also supported by an identical multidrug resistance profile (metronidazole, clindamycin, rifampicin) [11] and the same spectrum of associated mutations and acquired resistance genes identified in the UK ERR12670107.

The results are also a reminder to use appropriate media to test for metronidazole. Using of E-test on Brucella blood agar supplemented with 5 mg/L of haemin failed to detect metronidazole resistance in 11 (84.6%) of the 13 Polish *C. difficile* RT955 isolates. In addition, the EUCAST recommends Fastidious anaerobe agar medium, which revealed batch-dependent performance and failed to detect metronidazole resistance after 48 h of culture incubation 46.2% and 7.7% isolates. However, when we used Chocolate agar as a commercial ready-to-use haemin-enriched medium (10 g/L of haemoglobin) and BHI agar supplemented with 5, 10 and 20 mg/L of haemin, all isolates had increased MIC values after 24 and 48 h of culture between 4 and 12 mg/L (ECOFF ≥ 2 mg/L) and changed from the susceptible to the resistant category. The BHI medium supplemented with freshly prepared haemin was capable of detecting haem-dependent metronidazole resistance probably because it prevents haemin inactivation by prolonged incubation in light [30]. Although the Chocolate agar in our study was not freshly prepared, haem is present as a component of haemoglobin, which could provide haem more stability and suggests the Chocolate agar as an interesting commercially available alternative for haem-dependent metronidazole resistance testing.

The metronidazole resistance was encoded by T-to-G mutation (P*nim*B^G^) in the predicted promoter of the *nim*B gene (nt position −43) described by Oilatan et al. [17]. Except for the promotor, our isolates and the UK strain had two substitutions in the *nim*B gene, Tyr130Ser and Leu155Ile. The substitution *nim*B_p.Tyr130Ser has already been described as associated with metronidazole resistance in combination with a mutation in the *nim*B gene promoter in isolates from the MODIFY I and II studies [31]. The second, *nim*B_p.Leu155Ile, has been suggested as related to metronidazole resistance using predictive protein modelling [32], but this hypothesis was not supported by results from an expression assay under the tetracycline-inducible promoter (Ptet) [17]. The role of these mutations in metronidazole resistance should be further investigated.

Another newly described substitution in RT955 isolates is *rpo*B_p.Val134Ile, while *rpo*B_p.Arg505Lys has been previously described in RTs 027 and 176 [23,33,34]. The mutations in the *rpo*B gene can also be associated with resistance to fidaxomicin [35], but the isolates in our study were fidaxomicin-sensitive.

Our data show that RT955 is a multidrug-resistant ribotype with the potential of epidemic spread, as nine of the 13 Polish isolates were closely related to the British strain in wgMLST. Similar to Dingle et al., who investigated epidemic lineages of multidrug-resistant *C. difficile* [25], we found the co-occurrence of cephalosporin and fluoroquinolone resistance associated with the substitutions Thr82Ile in the GyrA and Val497Leu in the PBP3. Furthermore, a combination of metronidazole and fluoroquinolone resistance described by Olaitan et al. [17] in epidemic clade 2 isolates is also present in RT955.

This study is limited by its retrospective character, the lack of a uniform surveillance protocol and a limited number of participating hospitals and countries. A prospective surveillance study using a standardised surveillance protocol [36,37] is needed to estimate the current prevalence of RT955. Since the source of RT955 is unknown, the surveillance programme should not be restricted to hospital strains but should also include patients with CDI acquired outside healthcare facilities, as well as animal and environmental isolates. 

Of note, even though *C. difficile* RT 955 is a rare PCR ribotype and has only recently been identified, strains belonging to this ribotype but with different clinical, and microbiological characteristics have also been found retrospectively in Serbia in a CDI surveillance programme covering the period 2018 to 2022 (data not shown).

## Conclusions

Although RT955 belongs to ST1 (clade 2) and encodes binary toxin with characteristic deletions in the tcdC gene, commercial PCR assays targeting tcdC deletions may misclassify these strains as presumptive RT027. Therefore, PCR ribotyping remains essential for accurate identification of this ribotype, and binary toxin gene-positive isolates should be further characterised. Importantly, haem-dependent metronidazole resistance linked to nimB gene mutations, another marker for suspected RT955, is not reliably detected on Fastidious anaerobe agar; therefore, susceptibility testing should be performed using BHI agar supplemented with fresh haemin or commercial Chocolate agar. The identification of clonally related haeme-dependent metronidazole-resistant C. difficile RT955 in multiple hospitals indicates a need for prospective surveillance to estimate its prevalence in Europe.

## Data Availability

All raw sequence data in this study and the complete genome of strain 7908 have been submitted to the NCBI Sequence Read Archive (SRA) under accession number BioProject ID PRJNA1129291 and are listed in the Supplement.

## Supplementary Data

46000 Supplement
